# Optimization of Optical Machine Structure by Backpropagation Neural Network Based on Particle Swarm Optimization and Bayesian Regularization Algorithms

**DOI:** 10.3390/ma14112998

**Published:** 2021-06-01

**Authors:** Xinyong Zhang, Liwei Sun

**Affiliations:** 1 Shanghai Institute of Technical Physics, Chinese Academy of Sciences, Shanghai 200083, China; xyz9956@163.com; 2 University of Chinese Academy of Sciences, Beijing 100049, China; 3 Key Laboratory of Infrared Detection and Imaging Technology, Shanghai Institute of Technical Physics, Chinese Academy of Sciences, Shanghai 200083, China

**Keywords:** space camera, supporting structure, optical machine structure optimization, backpropagation neural network, particle swarm optimization, Bayesian regularization algorithm

## Abstract

Fit of the highly nonlinear functional relationship between input variables and output response is important and challenging for the optical machine structure optimization design process. The backpropagation neural network method based on particle swarm optimization and Bayesian regularization algorithms (called BMPB) is proposed to solve this problem. A prediction model of the mass and first-order modal frequency of the supporting structure is developed using the supporting structure as an example. The first-order modal frequency is used as the constraint condition to optimize the lightweight design of the supporting structure’s mass. Results show that the prediction model has more than 99% accuracy in predicting the mass and the first-order modal frequency of the supporting structure, and converges quickly in the supporting structure’s mass-optimization process. The supporting structure results demonstrate the advantages of the method proposed in the article in terms of high accuracy and efficiency. The study in this paper provides an effective method for the optimized design of optical machine structures.

## 1. Introduction

As an important part of the space camera’s optical machine structure, the optical machine structure greatly affects the imaging performance of the space camera’s mirror. To ensure the healthy working condition of the space camera ground assembly, ground testing, launch, and in-orbit operating conditions, multiple design criteria need to be considered to optimize and design complex mechanical structures such as optical machine supporting structure to meet the system performance indexes [1,2]. The system performance indicators mainly include light weight, stiffness, and strong resistance to external environmental interference [3,4]. Additionally, there may be interactions between different design criteria. During the launch process, the space camera will be greatly disturbed by random external vibrations. To avoid the space camera’s resonance during the launch process, the space camera’s optical machine system’s stiffness must be increased as much as possible [5]. Simultaneously, to reduce launch costs, the space camera’s mass needs to be reduced as much as possible while maintaining the imaging performance and lifetime of the camera [6].

With the continuous development of computer technology, finite element analysis (FEA) technology has been rapidly developed and widely used in many fields, including mechanical optimization and design [7,8,9,10,11]. In the optimized design of complex mechanical structures for finite element analysis, it is necessary to discretize the mechanical continuous structure into many units and a large number of calculations. Each finite element analysis will take a long time.

The Monte Carlo method (MCM), a numerical analysis of mathematical models [12], is a traditional method for optimizing mechanical structures by iterative finite element calculations with random sampling to continuously modify the model parameters [13,14,15,16]. The whole process requires a large number of samples and calculations, with high time and cost, and sometimes the optimization results have difficulty meeting the design accuracy requirements [17]. Therefore, it is necessary to introduce new optimization design methods for complex mechanical structures to improve optimization design efficiency and design accuracy. Since the function relationship between input random variables and output response in the optical machine structure optimization design has strong nonlinearity, it is difficult to solve the implied function relationship, so it is necessary to establish a numerical surrogate model to solve the above problems. In the numerical surrogate model, the functional relationship between the input random variables and the output random variables is represented by fitting a small batch of data samples [18].

Neural networks have been used to fit the nonlinear relationship between input variables and output responses in recent years due to their strong nonlinear fitting capabilities [19,20,21,22,23,24,25]. Neural networks have been applied in many fields, such as space-based large mirror structure, turbine disks, automotive bushings, etc. As a machine learning method, the backpropagation neural network (BPNN) has great advantages in fitting nonlinear functions [26,27]. BPNNs have a strong function approximation ability, which can be approximated to continuous functions with arbitrary accuracy. However, in training samples, BPNNs suffer from over-fitting problems, local optimum issues, slow late convergence, and poor generalization ability. These problems will affect their training efficiency and prediction accuracy [28].

The particle swarm optimization (PSO) algorithm proposed by Kennedy and Everhart is inspired by the social behavior of animals such as school of fish, insect swarms, and bird populations, ensuring the PSO algorithm has the abilities for global optimization [29,30,31]. The Bayesian regularization (BR) algorithm enables BPNN to avoid overfitting and improves the generalization of BPNN [32].

The purpose of this paper is to propose an efficient alternative model method named the backpropagation neural network method based on particle swarm optimization and Bayesian regularization algorithms (BMPB) to improve the computational efficiency and accuracy of fitting the implicit functions of nonlinear systems. In this method, the initial weight and initial threshold of ANNs are searched by the improved PSO algorithm; then, to obtain the trained neural network model, the BP algorithm training sample data determines the optimal weight and threshold. Finally, BMPB takes the optical machine system’s supporting structure as an example of the structural optimization design.

In the next parts of the paper, the basic theory of neural network, PSO algorithm, BR algorithm, and BMPB are introduced in Section 2; the specific use of BMPB is discussed in Section 3; BMPB is used in Section 4 to optimize the design of the supporting structure of the optical machine system; Section 5 is the conclusion of the paper.

## 2. Basic Theory

### 2.1. BPNN Method

**Figure 1:**
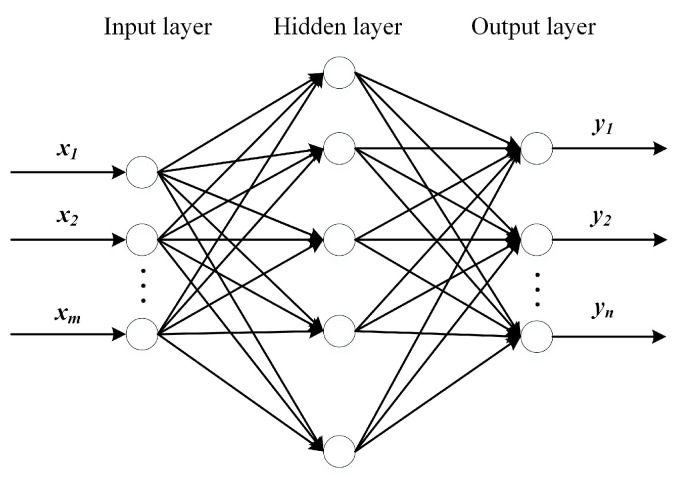
**The** BPNN model structure.

BPNN is a feed-forward neural network with strong nonlinear mapping capability. BPNN mainly consists of three parts: the input layer, hidden layer, and output layer. BPNN adopts the error backpropagation algorithm. Its training process mainly includes the forward propagation of the output result and the backpropagation of the error. The two steps are executed alternately, and the weight and threshold are constantly modified until the accuracy conditions are satisfied. The BPNN model is shown in Figure 1. The training process of BPNN replaces the traditional finite element analysis calculation process and avoids a large number of calculations, thus greatly improving the calculation efficiency.

### 2.2. PSO Algorithm

The PSO algorithm calculates each particle’s fitness by randomly initializing the particle swarm, updating the position and velocity of particles by counting the optimal value of the particle itself and the particle swarm, and searching for global optimization iterative process. The particle velocity update formula is as follows [18,29]:

(1) $$V_{i}\left(t+1\right){=\omega V}_{i}\left(t\right){+c}_{1}r_{1}\left[{\mathrm{Pbest}}_{i}\left(t\right){\;-\;X}_{i}\left(t\right)\right]{+c}_{2}r_{2}\left[\mathrm{Gbest}\left(t\right){\;-\;X}_{i}\left(t\right)\right]$$

where i is the particle number of the particle swarm, t is the number of iterations, $V_{i}\left(t+1\right)$ is the velocity of the ith particle in the t+1th iteration,  ω is the inertia weight, $V_{i}\left(t\right)$ is the velocity of the ith particle in the tth iteration, $c_{1}{,\;c}_{2}\;$ is the learning factors, ${\;r}_{1}{,\;r}_{2}$ are the random numbers between [0,1], ${\mathrm{Pbest}}_{i}\left(t\right)$ is the best position of the ith particle during t iterations, ${\;X}_{i}\left(t\right)$ is the position of ith particle in the tth iteration, ${\;\mathrm{and}\;\mathrm{Gbest}}_{i}\left(t\right)\;$ is the best position of the population in the tth iteration. The formula of the particle position update is as follows:

(2) $$X_{i}\left(t+1\right){=X}_{i}\left(t\right){+V}_{i}\left(t+1\right)$$

where $X_{i}\left(t+1\right)$ is the position of ith particle in the t+1th iteration.

The value of inertia weight ω greatly affects the global and local search capability of the PSO algorithm. A larger value of ω will lead to a stronger global search capability, while a smaller value of ω will lead to a weaker local search capability. Learning factors $c_{1}$ and $c_{2}$ enable particles to acquire the ability of self-learning and learning from group optimal particles. The suitable value of learning factors $c_{1}$ and $c_{2}$ can improve the optimization efficiency and avoid falling into the local optimal situation. In particle swarm optimization, the ideal state is to maintain strong global optimization ability in the early stage of optimization and strong local search ability in the late stage of optimization.

Therefore, to improve the PSO algorithm’s optimization ability, inertia weight ω, and learning factors $c_{1}$ and $c_{2}$ are adjusted to be dynamically updated with the number of iterations, and the modified formula is as follows:

(3) $$\omega \left(t\right){=\omega }_{\mathrm{ini}}+\sin\left(\frac{\pi t}{2t}\right){(\omega }_{\mathrm{fin}}{\;-\;\omega }_{\mathrm{ini}})$$

(4) $$c_{1}\left(t\right){=c}_{1\mathrm{ini}}\;-\;\sin\left(\frac{\pi t}{2t}\right){(c}_{1\mathrm{ini}}{\;-\;c}_{1\mathrm{fin}})$$

(5) $$c_{2}\left(t\right){=c}_{2\mathrm{ini}}\;-\;\sin\left(\frac{\pi t}{2t}\right){(c}_{2\mathrm{fin}}{\;-\;c}_{2\mathrm{ini}})\;$$

where ω(t) is the inertia weight in the tth iteration, T is the maximum number of iterations, $\omega_{\mathrm{ini}}$ is the initial value of the inertia weight, $\omega_{\mathrm{fin}}$ is the final value of the inertia weight in the Tth iteration, $c_{1}\left(t\right)$ and ${\;c}_{2}\left(t\right)$ are learning factors in the tth iteration, $c_{1\mathrm{ini}}$ and $c_{2\mathrm{ini}}$ are initial learning factors in the first iterations, and $c_{1\mathrm{fin}}$ and $c_{2\mathrm{fin}}$ are final learning factors in the last iterations.

### 2.3. BR Algorithm

The BR algorithm can avoid overfitting of BPNN and improve the generalization ability of BPNN by adjusting the objective function of BPNN. The objective function of the BPNN can be adjusted as follows [32]:

(6) $${F=\alpha E}_{w}{+\beta E}_{D}\;$$

where F is the objective function of the neural network, $E_{D}$ is the error sum of squares between the predicted value and the real value of the neural network training set, $E_{w}$ is the regularizer, and α and β are the regularization coefficients.

### 2.4. BMPB

As described above, BMPB is proposed to fit the nonlinear function between input variables and output responses in the optimization design of optical-machine structural parameters by fusing the improved PSO algorithm, BR algorithm, and BPNN. BMPB mainly consists of two parts. Firstly, an improved PSO algorithm is used to optimize the initial weight and threshold of BPNN. Then BPNN and the BR algorithm are used to train the samples to obtain the high-precision fitting functions of input variables and output variables. BMPB has the following advantages: (1) BMPB can fit the nonlinear relationship between the input random variable and the output response well; (2) BMPB only considers the relationship between input variables and output responses, which avoids a large number of finite element calculations, greatly reducing the time cost; (3) BMPB has a high prediction accuracy; (4) The improved PSO algorithm can ensure the diversity of the particle swarm and avoid BPNN falling into the local optimal. (5) Introducing the BR algorithm enables BPNN to avoid overfitting and improves the generalization of BPNN. (6) BMPB has a fast convergence speed and high efficiency.

## 3. Optimal Design Process of an Optical Machine Structure 

**Figure 2:**
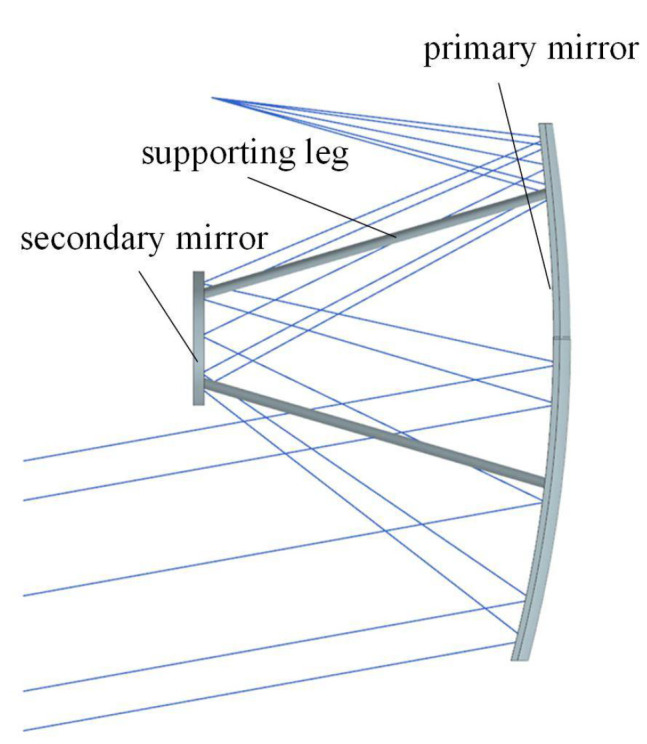
The schematic diagram of an optical system.

During the ground manufacturing phase and the ground assembly phase, the mirror surface shape of the space camera and the relative positions between the different mirrors are affected by gravity, which ultimately affects the imaging performance. In addition, space cameras are subjected to random vibrations during the launch phase, which may result in resonance. In order to ensure the excellent optical performance of the space camera, it is crucial to optimize the optical machine structure. The diagram of the optical system is shown in Figure 2. Maintaining a high stiffness of the optical structure can reduce the influence of gravity on the imaging performance of the space camera and avoid the resonance of the space camera during the rocket launch process. In order to reduce the launch cost, the space camera needs to minimize the space camera’s mass on the basis of ensuring the excellent imaging performance and lifetime of the space camera.

### 3.1. Finite Element Calculation and Integrated Analysis

**Figure 3:**
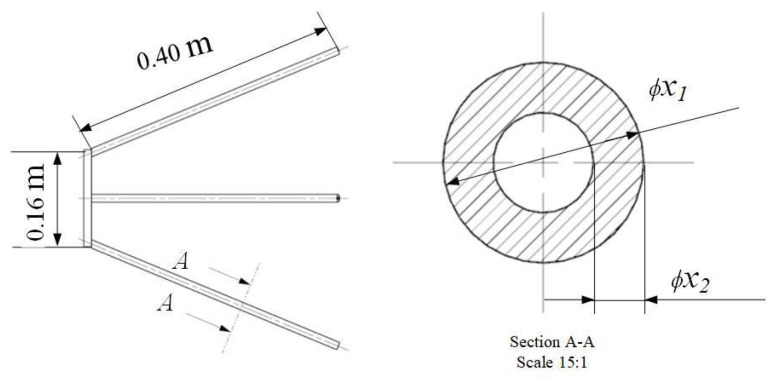
Secondary mirror supporting structure.

As an important component of a space camera, the supporting structure of the secondary mirror consists of a supporting ring and four supporting legs. In order to ensure the imaging performance and light weight of the space camera, the four supporting legs of the supporting structure need to be optimized. The simplified diagram of the secondary mirror supporting structure is shown in Figure 3. The outer diameter and wall thickness of each supporting leg are $x_{1}$ and $x_{2}$, respectively.

Firstly, the dimension parameters of the supporting structure of the secondary mirror are extracted for parametric modeling, and the 3D model is obtained. The finite element method is then used to divide the 3D model into grids, assign materials, and impose boundary constraints. Finally, the first-order modal frequency (H1) of the supporting structure of the secondary mirror is obtained by modal analysis.

The Latin Hypercube Sampling method was used to sample the secondary mirror’s supporting structure’s dimension parameters to obtain 400 data samples. The integrated analysis and design method were used to carry out the data samples’ finite element calculation to obtain training samples.

### 3.2. Training Neural Network Model

The training samples were randomly divided into a training set and a test set. BMPB was used to train the training set. After obtaining the optimal weight and threshold, the test set was tested to verify the prediction accuracy.

### 3.3. Optimization of Optical Machine Structure Performance Index

Firstly, the optimization objectives are confirmed and constraints are set. Then the improved PSO algorithm is used to optimize the trained neural network model to obtain the best optimization results.

### 3.4. Optimization Flow Chart 

**Figure 4:**
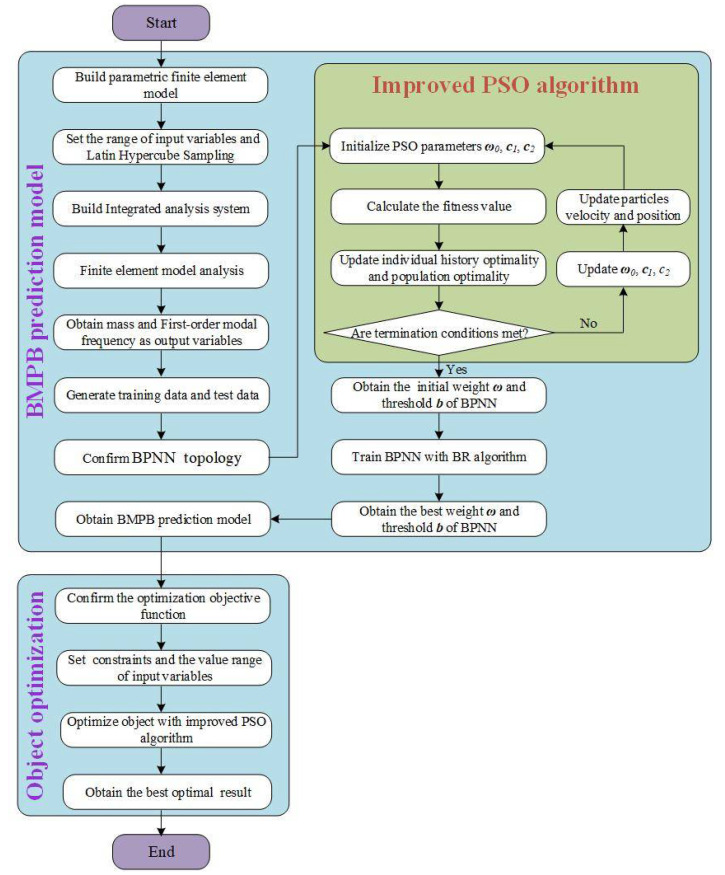
The optimization flow chart.

The optimization flow chart is shown in Figure 4. The optimization flow chart is mainly divided into two major parts. The first part is to build the BMPB prediction model; firstly, the integrated analysis system is used to obtain the training samples and test samples of BPNN, then the improved PSO algorithm is used to optimize the weight and threshold of BPNN, and finally, the BR algorithm is used to train the BPNN to obtain the BMPB prediction model. The second part is objective optimization, which firstly determines the objective function, then sets the constraints and input variable ranges, and finally uses the improved PSO algorithm to optimize the objective to obtain the best optimization results.

## 4. Optimal Design of Supporting Structure

The optical machine structure is subjected to gravity during the ground test phase, vibration impact during the launch phase, and temperature change during the in-orbit operation phase. The supporting structure’s function is to ensure the position accuracy of the mirror, thereby ensuring the high image quality of the mirror. BMPB is used to optimize the supporting structure’s dimensional parameters to meet the design requirements of light weight and high stiffness of the supporting structure.

### 4.1. Preliminary Preparation

**Figure 5:**
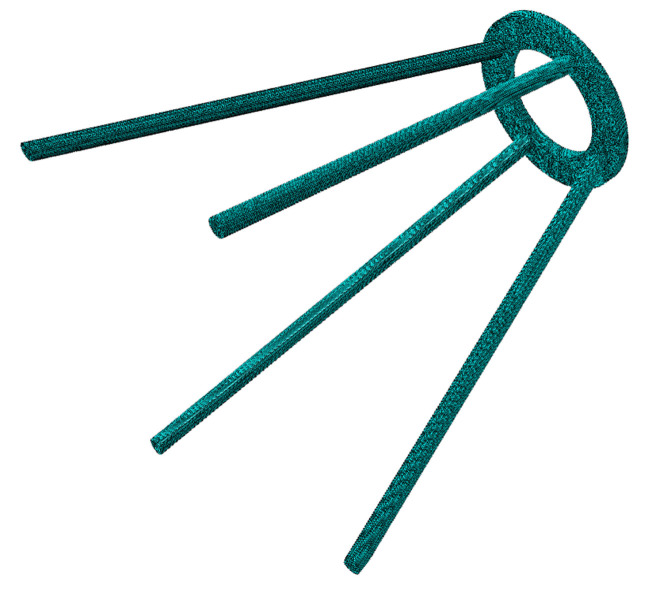
Finite element model of the supporting structure.

A parameterized method is used to establish a finite element model of the supporting structure. Aluminum_6061 was chosen as the material for the model, which has a modulus of elasticity of 68.9 Gpa and a Poisson’s ratio of 0.33. The finite element model is shown in Figure 5. Considering the influence of design variables on the output response quality and modal frequency. The outer diameter $x_{1}$ and the wall thickness $x_{2}$ of the supporting structure are used as input random variables. The input random variables are independent of each other. Table 1 shows the value range of the input random variables. The following formula can express the output response mass and the H1:

(7) $$F\left(x\right){=f(x}_{1}{,x}_{2})$$

(8) $$G\left(x\right){=g(x}_{1}{,x}_{2})$$

where F(x) and ${f(x}_{1}{,x}_{2})$ are the objective function of mass, and G(x) and ${g(x}_{1}{,x}_{2})$ are the objective function of H1.

### 4.2. Integrated Analysis

**Figure 6:**
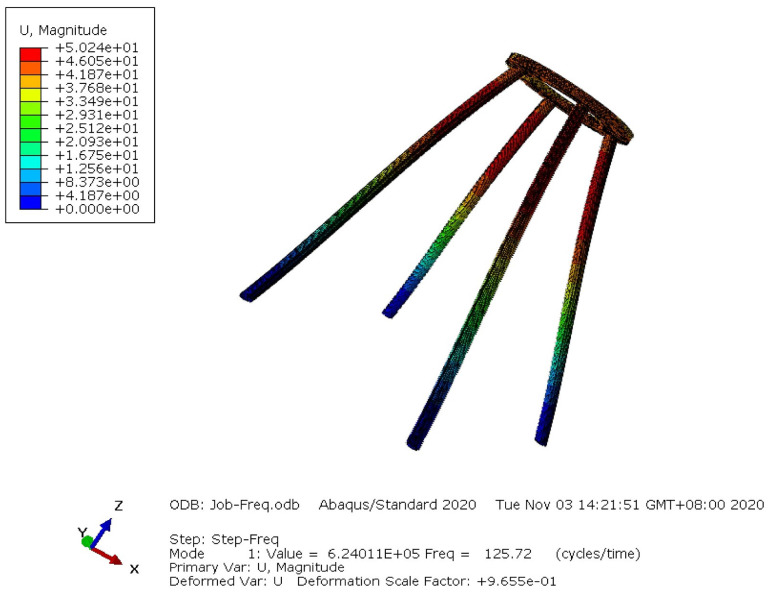
The first-order mode shape of the supporting structure.

The input random variables are sampled by the Latin Hypercube Sampling method to obtain 400 sets of input variable data. Since different tools and software are needed from modifying the dimensional parameters of the four supporting legs to finite element analysis, manual modification of finite element model parameters and finite element analysis are costly. The application of an integrated analysis platform can greatly improve efficiency. Input variable data are substituted into the finite element model for modal analysis, and 400 groups of output responses are obtained. Figure 6 shows the first-order mode shape of the supporting structure.

### 4.3. Build the BMPB Model

First, 320 sets of training samples and 80 sets of test samples are generated based on 400 sets of input and output data. Then the structure of the neural network is determined, the prediction error is set, and the BMPB prediction model is developed using the improved PSO algorithm, BR algorithm, and BPNN method in Section 2. The training sample data and the test sample data are pre-processed by normalization. The number of hidden neurons in BPNN is nine and the number of particles in the improved PSO algorithm is 40. Finally, the complex nonlinear implicit function relationship between the input variables and the supporting structure’s output responses is obtained.

**Figure 7:**
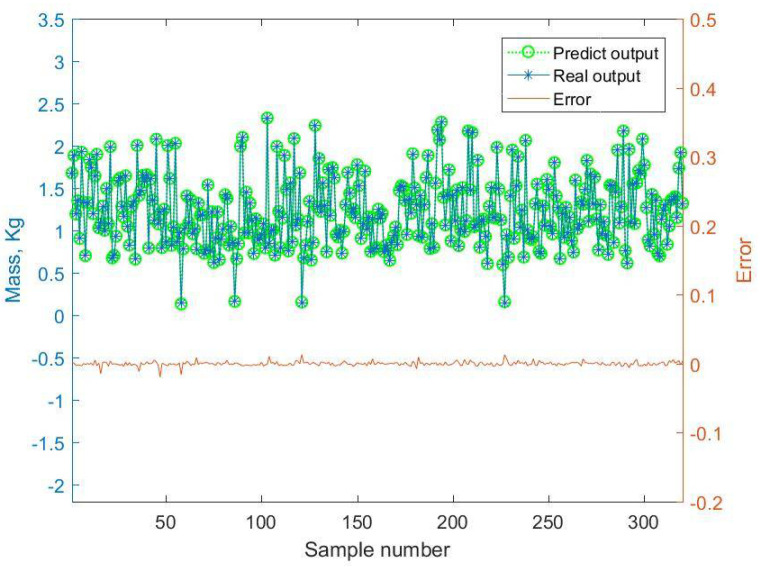
Prediction result of the mass’s training set.

**Figure 8:**
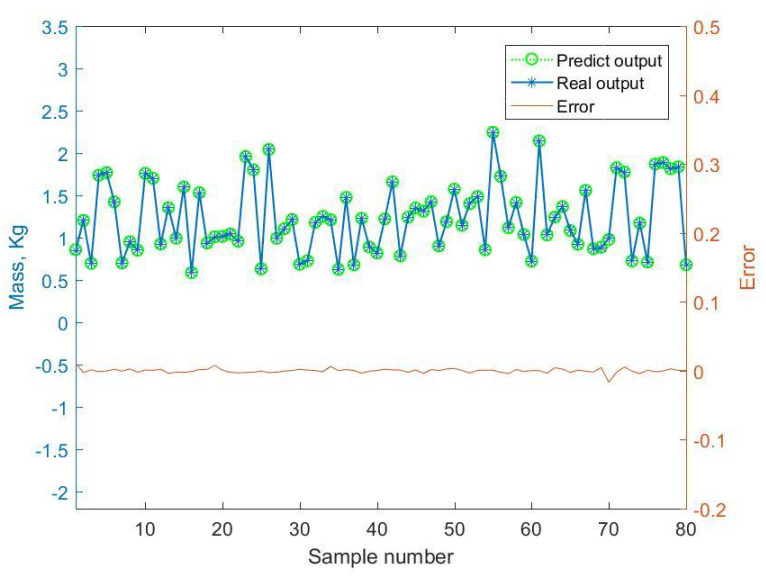
Prediction result of the mass’s test set.

By comparing the mass predictive results of the BMPB prediction model with the actual output response, the prediction results of the BMPB prediction model are obtained, and are shown in Figure 7 and Figure 8. The indicators for evaluating each effect of the BMPB prediction model are shown in Table 2, mainly including mean absolute error (MAE), mean squared error (MSE), root mean squared error (RMSE), and mean predictive accuracy (MPA).

**Figure 9:**
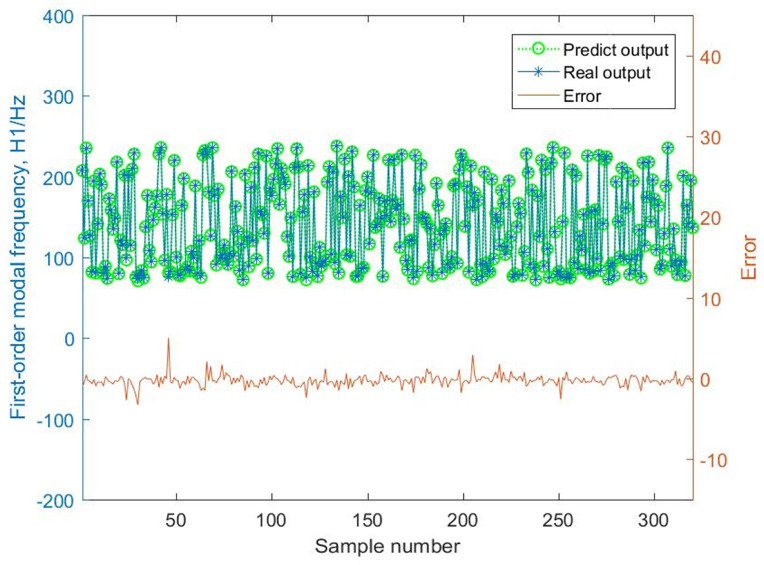
Prediction result of the first-order modal frequency’s training set.

**Figure 10:**
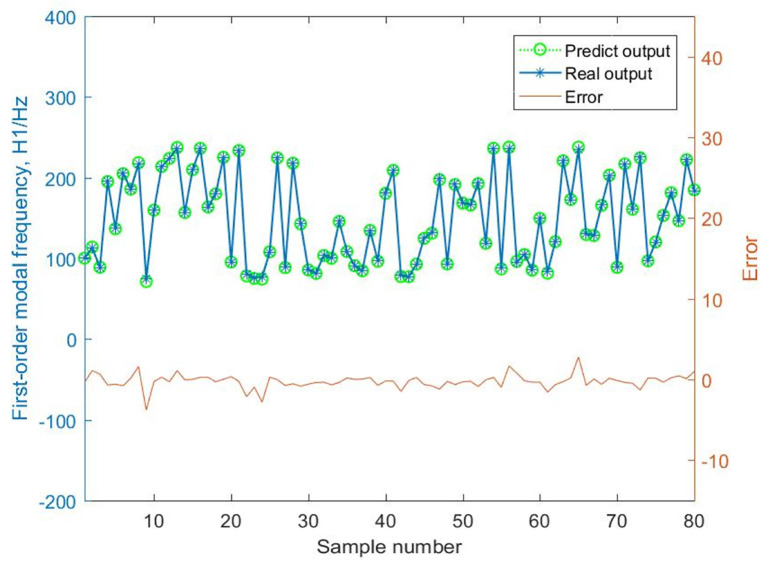
Prediction result of the first-order modal frequency’s training set.

Similarly, the BMPB prediction model’s results and evaluation metrics for the first-order modal frequency H1 of the supporting structure are shown in Figure 9 and Figure 10, and Table 3.

**Figure 11:**
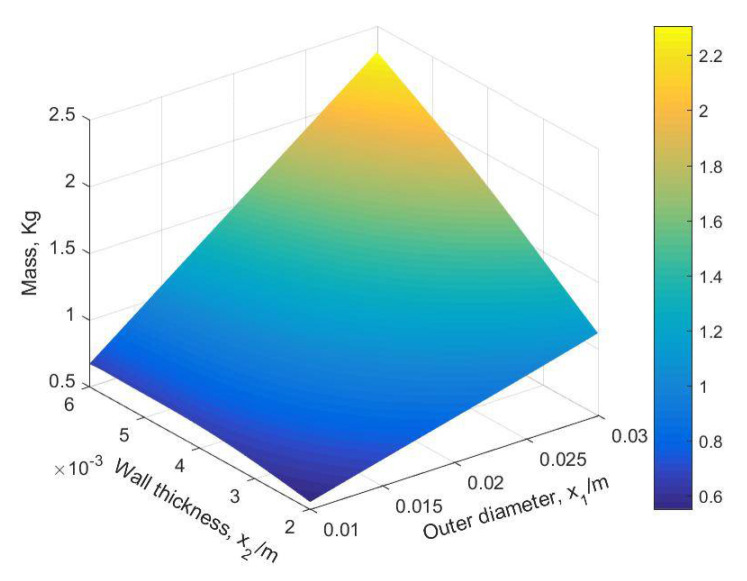
The predictive cloud picture of mass.

**Figure 12:**
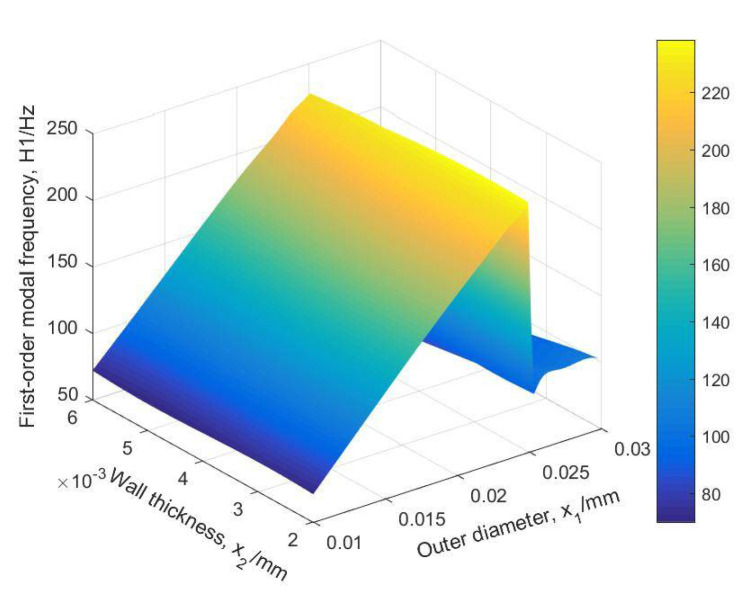
The predictive cloud picture of first-order modal frequency.

The predictive cloud pictures of mass and first-order modal frequency are shown in Figure 11 and Figure 12.

### 4.4. The Optimization of Supporting Structure Mass

To reduce the launching cost, it is necessary to reduce the supporting structure’s mass, so the mass of the supporting structure is taken as the optimization target. Simultaneously, to ensure the structure’s performance with high stiffness, the first-order frequency H1 of the supporting structure is taken as the constraint condition, and the mathematical model is as follows:
$\mathrm{Find}\;x={\left(x_{1}{,\;x}_{1}\right)}^{T}$min F(x)(9)$$\mathrm{s.t.}\{\begin{array}{l} G\left(x\right)\geq 150\mathrm{Hz}\\ 0.01\;m\leq x_{1}\leq 0.03\;m\\ 0.002\;m\leq x_{2}\leq 0.006\;m \end{array}$$


**Figure 13:**
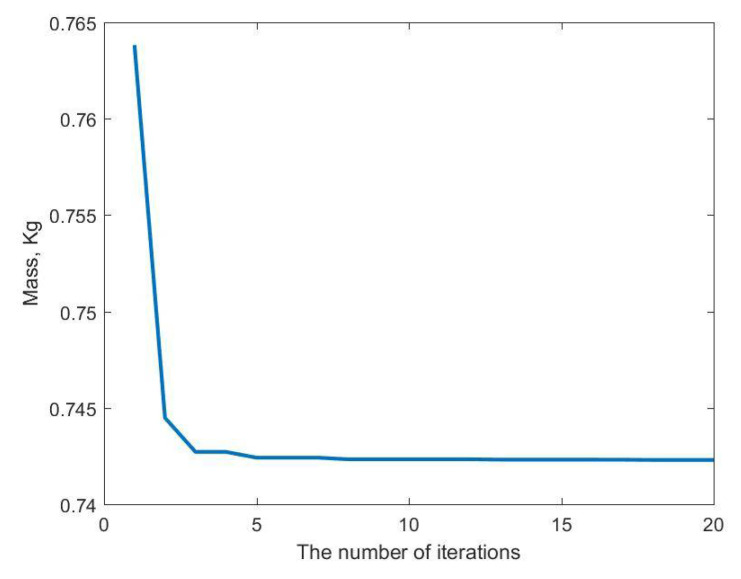
The optimization iterative process of the objective function.

The improved dynamic PSO method was used to optimize the objective function F(*x*), and the final optimization result was obtained after 20 iterations of searching. The iterative optimization process is shown in Figure 13 and the final optimization results are shown in Table 4.

### 4.5. Analysis

The BMPB prediction model proposed in this paper has a high prediction accuracy. As shown in Figure 7 and Figure 8 and Table 2, the training and test sets of the mass of the supporting structure were well predicted, with a mean absolute error of 2.2127 × 10^−3^ kg and 2.1471 × 10^−3^ kg, mean squared error of 1.0489 kg^2^ and 1.0429 kg^2^, root mean squared error of 3.2387 × 10^−3^ kg and 3.2293 × 10^−3^ kg, and mean predictive accuracy of 99.73% and 99.81%, respectively. The high mean predictive accuracy of both the mass’s training and test sets demonstrates the high prediction accuracy of the prediction model and the absence of overfitting. As shown in Figure 11, the mass of the supporting structure increases with increasing outer diameter $x_{1},$ and wall thickness $x_{2}$, and in addition, the change in external diameter has a large effect on the change in mass. As shown in Figure 9 and Figure 10 and Table 3, the training and test sets of the first-order modal frequency of the supporting structure were well predicted, with mean absolute error of 0.5368 Hz and 0.6078 Hz, mean squared error of 0.5943 Hz^2^ and 0.8135 Hz^2^, root mean squared error of 0.7709 Hz and 0.9019 Hz, and mean predictive accuracy of 99.55% and 99.49%, respectively. The high mean predictive accuracy of both the first-order modal frequency’s training and test sets demonstrates the high prediction accuracy of the prediction model and the absence of overfitting. As shown in Figure 12, the variation of wall thickness $x_{2}$ has little effect on the first-order modal frequency of the supporting structure, which first increases and then decreases sharply with the increase of the outer diameter $x_{1}$. By building a BMPB prediction model, a large number of finite element calculations can be avoided during the subsequent optimization of the optical machine structure, thus saving optimization time. When the input parameters of the optical machine structure change, the corresponding output response can be quickly predicted based on the BMPB prediction model that has been trained.

**Figure 14:**
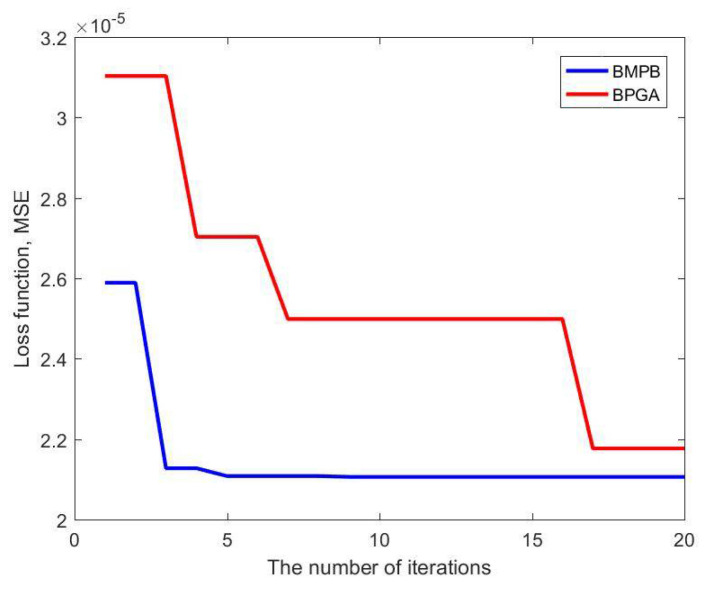
Comparison of BMPB and BPGA on the speed of convergence of the loss function.

The BMPB prediction model proposed in this paper improves the optimization efficiency by improving the PSO algorithm. The optimization of the supporting structure is shown in Table 4. The values of the outer diameter $x_{1}$ and the wall thickness $x_{2}$ are 0.0168 m and 0.002 mm, respectively, and the mass of the supporting structure is 0.7422 kg. Table 5 presents the comparison results between the MCM, BPGA and BMPB. They are all calculated on the same computer. During the optimization of the supporting structure using MCM, 400 simulations were performed in 1.04 × 10^5^ s. Optimization of the support structure using BPGA took 476 s. Optimization of the support structure using BMPB took 56 s. It can be seen that the BMPB has the shortest optimization time and optimizes the lightest weight. The accuracy of the BMPB’s optimization results is very close to the true value. As shown in Figure 13, BMPB reaches full convergence after the 5th iteration of the optimization search process and converges quickly. In conclusion, BMPB can greatly improve optical machine structure optimization’s computational accuracy and optimization efficiency. In addition, Figure 14 shows the comparison between BMPB and the backpropagation neural network based on a genetic algorithm (BPGA) regarding the speed of convergence of the loss function during the training of the BPNN model. The mean square error between the predicted output of the training samples and the real output of the training samples is chosen as the loss function. It can be seen from Figure 14 that BMPB converges faster than BPGA.

## 5. Conclusions

BMPB is developed to fit the nonlinear function between the input variable and the output response in the optical machine structure’s optimal design. Furthermore, the applicability of the proposed method is verified using the example of a supporting structure. Some conclusions of this paper are summarized as follows.

(1) The BMPB introduces the improved PSO algorithm into BPNN, which effectively improves the convergence efficiency of BPNN and avoids falling into the local optimum.

(2) The BMPB integrates the BR algorithm into BPNN, which enables BPNN to avoid overfitting and improves the generalization of BPNN;

(3) The supporting structure’s final optimization result is verified by finite element calculation, and the prediction accuracy is up to 99.4%.

(4) The BMPB can fit the nonlinear function between the input variable and the output response in the optimal design of the optical machine structure, with high prediction accuracy and the reduction of a lot of finite element calculation time.

We provided a way to optimize the design of optical and mechanical structures. This paper proposes the application of BMPB in the field of optimal design of optical machine structures, and the BMPB prediction model has the advantages of high prediction accuracy and avoidance of overfitting. It is expected that BMPB will play a great role in improving the process of performing complex multi-physics field analysis (such as gravity field analysis and thermal field analysis, etc.) for optical machine structures.

## Figures and Tables

**Table 1:** The distribution range of the input random variables.

Random Variables	Max, m	Min, m
$x_{1}$	0.03	0.010
$x_{2}$	0.006	0.002

**Table 2:** Mass predictive model evaluation indicators.

Evaluation Indicators	MAE, kg	MSE, kg^2^	RMSE, kg	MPA
Training samples	2.2127 × 10^−3^	1.0489 × 10^−5^	3.2387 × 10^−3^	99.73%
Test samples	2.1471 × 10^−3^	1.0429 × 10^−5^	3.2293 × 10^−3^	99.81%

**Table 3:** H1 predictive model evaluation indicators.

Evaluation Indicators	MAE, Hz	MSE, Hz^2^	RMSE, Hz	MPA
Training samples	0.5368	0.5943	0.7709	99.55%
Test samples	0.6078	0.8135	0.9019	99.49%

**Table 4:** Final optimization results.

$x_{1}$	$x_{2}$	Mass
0.0168 m	0.002 m	0.7422 kg

**Table 5:** Comparison of optimization results between the MCM and the BMPB.

Method	Optimization Time	Optimization Result	Accuracy
MCM	1.04 × 10^5^ s	0.7532 kg	100%
BPGA	476 s	0.8850 kg	91.4%
BMPB	56 s	0.7422 kg	99.4%

## Data Availability

The data presented in this study are available on reasonable request from the corresponding author.

## Abbreviations

BMPB	backpropagation neural network method based on particle swarm optimization and Bayesian regularization algorithms
FEA	finite element analysis
MCM	Monte Carlo method
BPNN	backpropagation neural network
PSO	particle swarm optimization
BR	Bayesian regularization
BPGA	backpropagation neural network based on genetic algorithm

## References

[1] Wei L., Zhang L., Gong X., Ma D.-M. (2017). Design and optimization for main support structure of a large-area off-axis three-mirror space camera. Appl. Opt..

[2] Gan X. Design for main support of long focal length space camera. Proceedings of the 2011 International Conference on Mechatronic Science, Electric Engineering and Computer (MEC).

[3] Zhu C., Xu T., Liu S., Bo Y., Liu Y. Design of primary mirror supporting structure and lightweight of space camera. Proceedings of the SPIE—The International Society for Optical Engineering.

[4] Yu F., Xu S. (2020). Support structure and optical alignment technology of large-aperture secondary mirror measured by back transmission method. Optik.

[5] Xia S., Shi J., Ren G., Li C. (2019). Calibration Mechanism Design and Stiffness Analysis. IOP Conf. Ser. Mater. Sci. Eng..

[6] Wu J. (2020). Design of High-lightweight Space Mirror Component Based on Automatic Optimization. J. Phys. Conf. Ser..

[7] Han Y.-Y., Zhang Y.-M., Han J.-C., Zhang J.-H., Yao W., Zhou Y.-F. (2006). Design and finite element analysis of lightmass silicon carbide primary mirror. Trans. Nonferrous Met. Soc. China.

[8] Lin Y.C., Lee L.J., Chang S.T., Cheng Y.C., Huang T.M. (2011). Numerical and Experimental Analysis of Light-Weighted Primary Mirror for Cassegrain Telescope. Appl. Mech. Mater..

[9] Williamson M.P. (1985). Finite-element analysis. Comput. Aided Eng. J..

[10] Zhu Y., Zhang Y., Shi J. (2021). Finite element analysis of flexural behavior of precast segmental UHPC beams with prestressed bolted hybrid joints. Eng. Struct..

[11] Meguid S.A., Kanth P.S., Czekanski A. (2000). Finite element analysis of fir-tree region in turbine discs. Finite Elem. Anal. Des..

[12] Tmr A., Dm A., Fab C., Oduj A., Jdc A., Bhj D., Dan S., Jam A., Jdm A. (2021). Simulation of Powder Bed Metal Additive Manufacturing Microstructures with Coupled Finite Difference-Monte Carlo Method. Addit. Manuf..

[13] Nakonechnyi A.N., Shpak V.D. (1994). Adaptive optimization of the Monte-Carlo method. Cybern. Syst. Anal..

[14] Yu W., Cao Z., Au S.K. (2010). Efficient Monte Carlo Simulation of parameter sensitivity in probabilistic slope stability analysis. Comput. Geotech..

[15] Guggenberger J., Grundmann H. (2004). Monte Carlo simulation of the hysteretic response of frame structures using plastification adapted shape functions. Probabilistic Eng. Mech..

[16] Asri Y.M., Azrulhisham E.A., Dzuraidah A.W., Shahrir A., Shahrum A., Azami Z. (2011). Fatigue life reliability prediction of a stub axle using Monte Carlo simulation. Int. J. Automot. Technol..

[17] Song L.-K., Bai G.-C., Fei C.W. (2019). Probabilistic LCF life assessment for turbine discs with DC strategy-based wavelet neural network regression. Int. J. Fatigue.

[18] Lks A., Cwfa B., Jie W.A., Gcb A. (2017). Multi-objective reliability-based design optimization approach of complex structure with multi-failure modes—ScienceDirect. Aerosp. Sci. Technol..

[19] Wang Z., Zhang J.A., Wang J.C., He X.A., Fu L.A., Tian F.A., Liu X.A., Zhao Y.A. (2019). A Back Propagation neural network based optimizing model of space-based large mirror structure. Optik.

[20] Wang D., Zhang S., Tan F., Zhi X., Zhen R. A method on lightweight for the primary mirror of large space-based telescope based on neural network. Proceedings of the International Symposium on Optoelectronic Technology & Application: Imaging Spectroscopy & Telescopes & Large Optics.

[21] Charytoniuk W., Chen M.S. (2000). Short-term load forecasting using Artificial Neural Networks. A review and evaluation. IEEE Trans. Power Syst..

[22] Magalhaes F.C., Ventura C., Abrao A.M., Denkena B., Meyer K. (2019). Prediction of surface residual stress and hardness induced by ball burnishing through neural networks. Int. J. Manuf. Res..

[23] Jung Y.W., Kim H.K. (2020). Prediction of Nonlinear Stiffness of Automotive Bushings by Artificial Neural Network Models Trained by Data from Finite Element Analysis. Int. J. Automot. Technol..

[24] Al-Garni A.Z., Jamal A., Ahmad A.M., Al-Garni A.M., Tozan M. (2006). Neural network-based failure rate prediction for De Havilland Dash-8 tires. Eng. Appl. Artif. Intell..

[25] Panagiotis A., Panayiotis R., Maria D. (2017). Feed-Forward Neural Network Prediction of the Mechanical Properties of Sandcrete Materials. Sensors.

[26] Zhao Q., Liu Q., Cao N., Guan F., Wang H. (2020). Stepped generalized predictive control of test tank temperature based on backpropagation neural network. AEJ Alex. Eng. J..

[27] Feng G., Lei S., Gu X., Guo Y., Wang J. (2021). Predictive control model for variable air volume terminal valve opening based on backpropagation neural network—ScienceDirect. Build. Environ..

[28] Jin C., Jin S.W., Qin L.N. (2012). Attribute selection method based on a hybrid BPNN and PSO algorithms. Appl. Soft Comput..

[29] Kennedy J. Particle swarm optimization. Proceedings of the ICNN’95—International Conference on Neural Networks.

[30] Gomes H.M. (2011). Truss optimization with dynamic constraints using a particle swarm algorithm. Expert Syst. Appl..

[31] Xu L., Huang C., Li C., Wang J., Wang X. (2021). Estimation of tool wear and optimization of cutting parameters based on novel ANFIS-PSO method toward intelligent machining. J. Intell. Manuf..

[32] Cawley G.C., Talbot N. (2007). Preventing Over-Fitting during Model Selection via Bayesian Regularisation of the Hyper-Parameters. J. Mach. Learn. Res..

